# Retracted: Recent Advances in DENV Receptors

**DOI:** 10.1155/2013/235198

**Published:** 2013-11-18

**Authors:** The Scientific World Journal

This paper has been retracted as it is found to contain a substantial amount of material from a number of previously published papers. The three most plagiarized papers are (1) K. I. Hidari and T. Suzuki, “Dengue virus receptor,” Tropical Medicine and Health, vol. 39, no. 4, supplement, pp. 37–43, 2011; (2) A. Cabrera-Hernandez and D. R. Smith, “Mammalian dengue virus receptors,” Dengue Bulletin, vol. 29, no. 662, pp. 119–135, 2005; (3) A. Cabrera-Hernandez, C. Thepparit, L. Suksanpaisan, and D. R. Smith, “Dengue virus entry into liver (HepG2) cells is independent of hsp90 and hsp70,” Journal of Medical Virology, vol. 79, no. 4, pp. 386–392, 2007 (see [1]).
